# Mortalité néonatale précoce et ses déterminants dans une maternité de niveau I à Yaoundé, Cameroun

**Published:** 2012-11-26

**Authors:** David Chelo, Francisca Monebenimp, Francine Romyale Tedongmeza Npanguepko, Félix Tietche

**Affiliations:** 1 Centre Mère et Enfant de la Fondation Chantal BIYA, Yaoundé, Cameroun; 2 Département de Pédiatrie, Faculté de Médecine et des Sciences Biomédicales de l'Université de Yaoundé 1, Cameroun; 3 Centre Hospitalier et Universitaire de Yaoundé, Cameroun; + Décédé le 16 Mai 2009

**Keywords:** mortalité, néonatale, précoce, déterminants, maternité, Cameroun, mortality, neonatal, early, determinant, maternity, Cameroon

## Abstract

**Introduction:**

Le but de ce travail était de déterminer les facteurs de risque de la mortalité néonatale dans une maternité de niveau I.

**Méthodes:**

Il s'agissait d'une étude de cohorte rétrospective qui s'est déroulée d'Août 2007 à Janvier 2008 au Centre d'Animation Sociale et Sanitaire (CASS) de Nkoldongo à Yaoundé. Tout nouveau-né accouché vivant à la maternité était recruté. Les données collectées à partir du dossier de la mère portaient sur l'histoire anté et perpartum, les paramètres anthropométriques du nouveau-né et sa survie après les trois jours de suivi. L'analyse des données a été faite avec le logiciel Epi Info version 3.5.1.

**Résultats:**

Huit décès ont été enregistrés sur les 1263 nouveau-nés enrôlés, soit un taux de mortalité néonatale précoce de 0,5%. Tous les décès sont survenus dans les 24 premières heures de vie. Les causes de décès étaient: l'asphyxie néonatale (6/8) et la prématurité (2/8). Le facteur de risque associé à la mortalité serait la rupture prolongée des membranes de plus de 12 heures (OR: 4,69; IC 95%: (0,95-23,09); p <0,05).

**Conclusion:**

La mortalité néonatale précoce est faible dans cette formation sanitaire de niveau I. L'asphyxie néonatale est la première cause de décès chez les nouveau-nés. La rupture prolongée des membranes corollaire à l'infection néonatale serait un facteur de risque associé à la mortalité néonatale. Par conséquent, le renforcement des capacités des personnels de santé est une nécessité en vue d'une prise en charge appropriée des parturientes.

## Introduction

La mortalité néonatale représente 40% des décès des enfants de moins de 5 ans dans le monde, en particulier dans les pays en voie de développement [1]. La réduction de la mortalité néonatale passerait par des programmes efficaces qui prendraient en compte des interventions sur les facteurs de risque modifiables. D'après un rapport du Ministère de la Santé Publique au Cameroun, 33% de parturientes ne fréquentent pas de formations sanitaires pour leurs consultations prénatales. Par ailleurs en ce qui concerne l'accouchement assisté par un personnel de santé, il existe une disparité entre les riches (88%) et les pauvres (29.5%) [2]. Les formations sanitaires de niveau I recrutent dans la majorité de cas des femmes enceintes à ressources financières limitées. La qualité des soins dans ces structures sanitaires est faiblement documentée. C'est ainsi que nous avons réalisé cette étude dont l'objectif était de déterminer le taux de mortalité néonatale et ses déterminants dans une formation sanitaire de niveau I à Yaoundé. Le but étant à terme d'élaborer des politiques spécifiques sur le renforcement des capacités des personnels de santé.

## Méthodes

Il s'agissait d'une étude transversale qui s'est déroulée du 1er Août 2007 au 31 Janvier 2008 au Centre d'Animation Sociale et Sanitaire (CASS) de Nkoldongo. Cette formation sanitaire confessionnelle catholique, située dans un quartier populaire de Yaoundé, possède une salle de travail de trois lits, une salle d'accouchement dotée de deux tables d'accouchement et une unité de suivi post partum de 33 lits. Les consultations prénatales et les accouchements sont assurés par 13 personnels infirmiers qualifiés. Les femmes enceintes présentant des facteurs de risque de mauvais pronostic étaient référés avant l'accouchement dans les structures sanitaires de niveau élevé. Un pédiatre vacataire assure des consultations néonatales deux fois par semaine. Une fois la mère contactée en salle de travail, le but et les explications sur l'étude lui étaient donnés en vue d'obtenir son consentement verbal. Les nouveau-nés accouchés vivants à la maternité du CASS étaient inclus dans l'étude de manière consécutive. Les données recueillies à partir de l'interrogatoire des mères, de leurs dossiers et des dossiers des nouveau-nés étaient consignées dans une fiche conçue à cet effet. Les informations relatives aux antécédents prénatals, au déroulement de l'accouchement, à l'examen physique du nouveau-né. Il s'agissait des caractéristiques démographiques, de l'âge gestationnel, du score d'Apgar, des paramètres anthropométriques et de la survie du nouveau-né. Les nouveau-nés étaient suivis pendant les trois jours que dure le séjour hospitalier dans ce Centre en cas d'accouchement normal.

L'analyse des données a été faite à l'aide du logiciel Epi Info version 3.5.1.Le taux de décès néonatals était notre critère de jugement. Les variables associées à la mortalité à p<0,07 à l'analyse univariée étaient incluses dans la régression logistique pour déterminer les facteurs qui lui sont indépendants. Les tests chi-carré ont été utilisés pour la comparaison des proportions et le test de Student pour la comparaison des groupes. Le niveau de significativité était établi pour une valeur p<0,05.

L'étude a été approuvée sur les aspects scientifiques et éthiques par le comité de recherche de la Faculté de Médecine et des Sciences Biomédicales de l'Université de Yaoundé 1.

## Résultats

Pendant la période d'étude, 1623 nouveau-nés ont été recrutés. Parmi eux, 834 (51,4%) étaient de sexe féminin tandis que 789 (48,6%) étaient de sexe masculin Nous avons dénombré 8 décès au cours des trois jours suivant l'accouchement. Le taux de décès néonatal était de 0,5%. Le Tableau 1 illustre les caractéristiques sociodémographiques de la population d'étude. Pour 1562 (96,3%) nouveau-nés, la grossesse était à terme alors que 61 (3,7%) étaient des accouchements prématurés. les nouveau-nés ayant un poids de plus de 2500g représentaient 1531 soit 94,3% de l'échantillon.

**Tableau 1:** Caractéristiques sociodémographiques des nouveau-nés recrutés à la maternité de niveau I, CASS, Août 2007 à Janvier 2008

Paramètres	Effectif	Pourcentage
**Sexe**		
Féminin	834	51,4
Masculin	789	48,6
**Age gestationnel (semaines)**		
< 37	61	3,7
≥37	1562	96,3
**Poids de naissance (grammes)**		
< 2500	92	5,7
≥2500	1531	94,3
**Age maternel (années)**		
<20	304	18,7
20-35	1232	75,9
>35	87	5,4
**Niveau d'instruction**		
Primaire et analphabète	359	22,1
Secondaire et supérieur	1264	77,9
**Profession des mères**		
Sans emploi	1016	63,6
Emploi rémunéré	607	36,4

La majorité des mères, 1232 au total (75,9%) avaient un âge compris entre 20 et 35 ans. Mille deux cents soixante-quatre (77,9%) avaient au moins un niveau d'instruction du secondaire. Les mères sans emploi comptaient pour 1016 (63,6%).

### Morbidité néonatale

Le processus morbide chez les nouveaux nés malades, listés dans le Tableau 2, était les suivants: la rupture prolongée des membranes chez 127 (70,9%) nouveau-nés (OR=11,59; 95% IC (8,11-16,48); p<0,0001), la fièvre chez la mère (OR=6,54; 95% IC (4,5-9,52); p<0,0001), la fièvre chez la mère après accouchement (OR 3,59; 95% IC (2,59-4,97); p< 0,0001), l'accouchement dystocique (OR=1,99; 95% IC (1,47-2,69); p<0,0001) et le liquide amniotique méconial à compléter p =0,012). Les nouveau-nés prématurés (OR=3,59; 95% IC 2,14-6,02); p<0,0001); et ceux ayant eu une asphyxie néonatale (OR=3,25; 95% IC (1,96-5,37), p<0,0001) étaient respectivement plus susceptibles d'être malades que les nouveau-nés à terme et ceux n'ayant pas eu de détresse respiratoire à la naissance. On peut relever que 66 (4,1%) nouveau-nés ont été transférés dans des services compétents de niveau élevé au cours des trois jours suivant la naissance. Les motifs de transfert étaient essentiellement 33 cas (2%) de suspicion d'infection néonatale, 18 (1,1%) de détresse respiratoire, 7 (0,4%) prématurés.

**Tableau 2:** Facteurs de morbidité chez les nouveau-nés recrutés à la maternité de niveau I, CASS, Août 2007 à Janvier 2008

Facteurs de risque	Nouveau-nés malades n= 399	Nouveau-nés non malades n= 1224	OR	95% IC	p
**Rupture prolongée des membranes**	**366**	**1187**	11,59	8,11 < OR < 16,48	<0,0001
Oui	127 (70,9%)	52 (29,1%)			
Non	239 (17,4%)	1135(82,6%)			
**Fièvre pendant le travail**	**352**	**1168**	6,54	4,5 < OR < 9,52	<0,0001
Oui	81(61,4%)	51(38,6%)			
Non	271(19,5%)	1117(80,5%)			
**Fièvre post accouchement**	**369**	**1206**	3,59	2,59 < OR < 4,97	<0,0001
Oui	82(48,0%)	89(52,0%)			
Non	287(20,4%)	1117(76,6%)			
**Accouchement**	**374**	**1204**	1,99	1,47 < OR < 2,69	0,0001
Dystocique	79(35,6%)	143(64,4%)			
Eutocique	295(21,8%)	1061(78,2%)			
**Liquide amniotique**	**373**	**1203**	1,32	1,04 < OR < 1,67	0,012
Méconial	168(26,7%)	461(73,3%)			
Clair	205(21,6%)	742(78,4%)			
**Age gestationnel**	**398**	**1220**	3,59	2,14 < OR < 6,02	<0,0001
<37semaines	32(52,5%)	29(47,5%)			
≥37 semaines	366(23,5%)	1192(76,3%)			
**Apgar 1 minute**	**399**	**1224**	3,25	1,96 < OR < 5,37	<0,0001
<7	30(50,0)	32(50,0)			
>7	367(23,5)	1192(76,5)			

### Mortalité néonatal

Huit nouveau-nés sont décédés dans la formation sanitaire pendant la période d'étude. Les causes de décès étaient l'asphyxie néonatale chez 6 nouveau-nés (75%) et la prématurité chez 2 autres (25%). Tous les nouveau-nés sont décédés dans les 24 premières heures de vie. Les facteurs de risque dans le Tableau 3 associés à la mortalité néonatale étaient: la rupture prolongée des membranes de plus de 12 heures (OR=4,96; IC 95% (1,17-20,98); P<0,05).

**Tableau 3:** Facteurs de risque associés à la mortalité chez les nouveau-nés recrutés à la maternité de niveau I, CASS, Août 2007 à Janvier 2008

Facteurs de risque	Nouveau-nés décédés N=8	Nouveau-nés vivants N=1615	OR	IC 95%	P
**Analyse univariée**					
**Age mère**			1,88	0,23-15,50	0,44
< 19 ans	1	106			
≥19 ans	7	1402			
**Age gestationnel**			4,16	(0,5-34,5)	0,2
<37 semaines	1 (2)	50 (98)			
≥37 semaines	7 (0,5)	1458 (99,5)			
**Fièvre mère**			4,96	1,17-20,98	0,04
Oui	2	121			
Non	5	1333			
**Rupture prolongée des membranes**			4,96	(1,17-20,98)	0,04
Oui	3 (1,18)	160 (98,2)			
Non	5 (0,4)	1325 (99,6)			
**Liquide amniotique méconial**			Indéfini	2,89 - indéfini	0,001
Oui	7	590			
Non	0	917			
**APGAR 5min**			Indéfini	110,4 - indéfini	0,0000
<7	7	7			
≥7	0	528			
**Poids naissance**				1,18-30,01	0,06
<2500g	2	8	5,92		
≥2500g	6	1431			
**Régression logistique**				
Fièvre maternelle			2,91	0,52-16,57	0,20
Rupture prolongée des membranes			4,69	0,95-23,09	0,05

## Discussion

Le but de notre travail était de déterminer les facteurs de risque de la mortalité néonatale dans une structure de niveau 1. Cette formation sanitaire accueille des femmes de faible niveau socio-économique constituant la grande majorité des parturientes. Sur les 1263 nouveau-nés enrôlés, huit décès ont été enregistrés. L'analyse univariée a montré que les facteurs de risque liés à la mortalité incluaient: la fièvre maternelle péripartale, le liquide amniotique méconial et le poids de naissance du nouveau-né inférieur à 2.500 g. Le seul facteur indépendant associé à la mortalité néonatale serait la rupture prolongée des membranes.

Dans cette structure sanitaire de niveau 1, les consultations prénatales ainsi que les accouchements sont conduits par un personnel infirmier formé à cet effet. Il n'existe pas de médecin pour prendre en charge la femme enceinte en dehors du pédiatre qui assure des consultations néonatales deux fois par semaine. L'évaluation clinique des nouveau-nés ainsi que leur orientation incombe à ce personnel dont la plupart (80%) n'a pas reçu la formation en soins obstétricaux et néonatals essentiels et d'urgence de l'Organisation Mondiale de la Santé (OMS) [3]. Aussi la pratique courante dans cette formation sanitaire est la référence immédiate, dans les cinq hôpitaux de niveau élevé de la ville, des femmes présentant un risque de complication lors de l'accouchement.

Les données de cette étude transversale étaient collectées à partir des dossiers de parturientes. Certaines informations spécifiques étaient manquantes et des définitions de variables n'étaient pas uniformes pour ces personnels qui prenaient en charge la mère et le nouveau-né si bien que des biais de mauvais classement pourraient expliquer pourquoi certains facteurs plausibles n'étaient pas des déterminants observés dans cette étude.

Le taux de décès néonatals était de 5%0 pour les trois premiers jours alors qu'en Afrique sub saharienne en 2009, ce taux était de 35,9%0 [4]. Il était de 27%0 au cours de la première semaine de vie dans un district rural du Burkina-Faso entre 2006 et 2007 [5]. Au Cameroun, il était de 31%0 au niveau national en 2011 pour toute la période néonatale [6]. Même en estimant que la mortalité néonatale des premières 24h de vie représente environ 50% de la mortalité néonatale globale [7, 8], le taux retrouvé dans notre étude reste relativement bas. Il est cependant plus élevé que le taux de 1,9 pour mille retrouvé par Baker et coll. [9] en colligeant toutes les naissances à New-York de 1991 à 2001. Dans notre série, le taux de mortalité est probablement sous estimé puisqu'il ne prend en compte que les nouveau-nés décédés in situ dans la formation sanitaire pendant les trois premiers jours de vie. En outre, la survie des 66 (4,1%) nouveau-nés transférés dans d'autres formations sanitaires de niveau élevé parce que nécessitant une assistance spécialisée pour infection néonatale, asphyxie néonatale et prématurité, n'avait pas été recherchée. Ces affections sont connues être responsables respectivement de 25%, 23% et de 29% de mortalité néonatale [7]. Par ailleurs, la politique de transfert in utéro systématique devant la reconnaissance par ce personnel de santé des grossesses à risque élevé pourrait indirectement contribuer au faible taux de décès. Aussi, les nouveau-nés décédés à domicile dans ce délai de trois jours étaient ignorés dans ce décompte. Au Pakistan, un taux de mortalité néonatale de 47%0 avait été retrouvé par Imtiaz et al [10]. Contrairement à notre travail, les parturientes étaient recrutées entre 20 et 26 semaines de grossesse. La mère et le nouveau-né étaient suivis dans les 48 heures post naissance et revus le 28e jour permettant ainsi de maximiser les statistiques de décès.

Tous les huit décès enregistrés sont survenus pendant les 24 premières heures de vie ce qui corrobore les trouvailles de certains auteurs qui ont montré que 50% de décès de nouveau-nés surviennent durant les premières 24 heures de vie et 75% dans la première semaine de vie [8]. Hafizur et al au Bangladesh [11] retrouvent 76% de décès dans les trois premiers jours de vie dont 37% lors des 24 premières heures.

Les causes de décès dans notre série étaient l'asphyxie néonatale (75%) et la prématurité (25%). Les trois principales causes directes de mortalité néonatale en Afrique sont par ordre de grandeur, la prématurité, l'infection néonatale et l'asphyxie néonatale [7]. Monebenimp et al dans une étude réalisée au CHU de Yaoundé retrouvent les causes de mortalité néonatale dans l'ordre sus-cité [12]. Selon Frøen et al les groupes de causes de mortalité périnatale dans 7 populations à travers le monde incluant deux populations dans les pays en développement seraient les évènements intrapartaux, les infections et les anomalies congénitales [13]. Tachiweyika et al [14] au Zimbabwe incriminent les complications du travail d'accouchement, les accouchements à domicile et la prématurité. Lawn et al [1] dans une méta-analyse incluant 46 études trouvent qu'en moyenne 23% de décès néonataux sont attribués à des causes intrapartales. Le Centre où l'étude a été effectuée accueille les populations parmi les plus démunies de la ville de Yaoundé. En cas de grossesse à haut risque ou d'accouchement difficile, il est proposé aux parturientes un transfert dans une structure ayant un meilleur plateau technique. Ces structures de niveau 2 ou 3 ont des coûts de soins bien plus élevés. Il est possible que des parturientes ayant accouché des enfants souffrant d'asphyxie néonatale aient été proposées pour un transfert, mais qu'elles aient refusé à cause de l'incidence financière. La pauvreté est en effet reconnue pour être associé à un risque élevé de mortalité néonatale [8].Nous n'avions pas d'argument irréfutables de diagnostic d'INN pendant la période de suivi des nouveau-nés. Mais la grande corrélation entre la rupture prolongée des membranes et les décès néonataux retrouvée dans notre travail peut suggérer la possibilité d'INN que nous n'avons pas eu l'occasion de prouver. De plus, tous les nouveau-nés avec histoire de RPM sont systématiquement adressés dans un service de néonatalogie de la place pour investigation d'une éventuelle INN.

La rupture prolongée des membranes de plus de 12 heures, corollaire de l'infection néonatale, était le facteur indépendant associé à la mortalité néonatale. La taille réduite de notre échantillon et le fait que le traçage de la survie des nouveau-nés transférés n'était pas observé constitueraient une des limites de notre étude. Ceci pourrait expliquer pourquoi certains facteurs potentiels et plausibles retrouvés à l'analyse univariée notamment la prématurité et l'asphyxie néonatale ne seraient pas être validés à la régression logistique. Dans notre Centre d'étude, le personnel infirmier avait été formé au suivi optimal des grossesses. L'OMS estime que les décès néonataux dépendraient en grande partie du lieu d'accouchement ainsi que du type de soins procurés pendant cet accouchement [7, 8]. La prévention et une meilleure prise en charge des pathologies de grossesse réduiraient considérablement la mortalité périnatale [15]. Aussi, la formation du personnel de santé dans le domaine contribuerait à cette réduction [16]. En Indonésie, Titaley et al [17] ont démontré que dans les régions où plus de 87,5% d'accouchements étaient assistés par un personnel de santé formé, la mortalité néonatale était réduite de 60% par rapport aux régions où cette assistance était de moins de 25%. En Zambie, Gill et al [18] montrent que le taux de mortalité néonatale dans un groupe de femmes assistées lors de leur accouchement par des accoucheuses traditionnelles formées était de 45% inférieur au groupe où les femmes étaient assistées par des accoucheuses non formées.

## Conclusion

Cette étude transversale a révélé un taux de décès des nouveau-nés de 0.5% dans les trois jours qui suivaient la naissance. La rupture prolongée des membranes ayant pour corollaire l'infection néonatale, serait un facteur indépendant lié à la mortalité néonatale. L'asphyxie néonatale est la première cause de décès chez les nouveau-nés.Il demeure qu'un accent particulier soit mis sur le renforcement des capacités du personnel pour l'amélioration de la qualité des consultations prénatales et la prise en charge de la mère et du nouveau-né lors de l'accouchement.
